# ATP13A2 (PARK9) protein levels are reduced in brain tissue of cases with Lewy bodies

**DOI:** 10.1186/2051-5960-1-11

**Published:** 2013-05-09

**Authors:** Karen E Murphy, Louise Cottle, Amanda M Gysbers, Antony A Cooper, Glenda M Halliday

**Affiliations:** Neuroscience Research Australia, Sydney, 2031 Australia; School of Medical Sciences, Faculty of Medicine, University of New South Wales, Sydney, 2052 Australia; The Garvan Institute of Medical Research, Sydney, 2010 Australia; St Vincent’s Clinical School, Faculty of Medicine, University of New South Wales, Sydney, 2052 Australia; School of Biotechnology and Biomolecular Sciences, Faculty of Science, University of New South Wales, Sydney, 2052 Australia; Neuroscience Research Australia, Barker Street, Randwick, 2031 Australia

**Keywords:** α-synuclein, ATP13A2, β-amyloid, Dementia with lewy bodies, PARK9, Parkinson’s disease, Protein levels

## Abstract

**Background:**

*ATP13A2* (*PARK9*) loss of function mutations are a genetic cause of an early-onset form of Parkinson’s disease (PD), with *in vitro* studies showing that ATP13A2 deficits lead to lysosomal and mitochondrial dysfunction and α-synuclein accumulation, while elevated ATP13A2 expression reduces α-synuclein toxicity. The three human brain tissue studies assessing changes in ATP13A2 expression in PD produced divergent results; mRNA is increased while protein levels were observed to be either increased or decreased. This apparent conflict in protein levels might have arisen from examining Lewy body disease cases with coexisting Alzheimer-type pathologies.

To assess whether ATP13A2 levels in Lewy body disease are modified by Alzheimer-type β-amyloid deposition, we evaluated cases of pure PD and pure dementia with Lewy bodies (DLB) for changes in ATP13A2, α-synuclein and β-amyloid protein levels in cortical regions with and without Lewy bodies.

**Results:**

In all Lewy body disease cases, we identified decreased ATP13A2 protein levels that correlated with increases in both α-synuclein and β-amyloid. Partial colocalization was observed between ATP13A2 and α-synuclein in Lewy bodies, whereas ATP13A2 did not colocalize with pathological β-amyloid deposition.

**Conclusions:**

Our data show that patients with Lewy body diseases have an overall deficit in ATP13A2 protein levels, with the remaining protein being more insoluble and partially redistributing towards Lewy bodies. This supports the concept that increasing ATP13A2 levels may offer potential therapeutic benefits to patients with Lewy body diseases.

**Electronic supplementary material:**

The online version of this article (doi:10.1186/2051-5960-1-11) contains supplementary material, which is available to authorized users.

## Background

Autosomal recessive loss-of-function mutations in the gene *ATP13A2* (also designated *PARK9*) are causative for early onset Kufor-Rakeb syndrome [1], an autosomal recessive juvenile onset form of L-dopa-responsive parkinsonism that exhibits clinical features of Parkinson’s disease (PD). ATP13A2 is highly expressed in the human brain with greatest expression observed in the substantia nigra pars compacta, a region that displays progressive degeneration of dopamine neurons in PD [1]. ATP13A2 is a P-type ATPase that is predicted to function as a cation metal transporter [2], and is localized in acidic membrane compartments thought to be lysosomes [1, 3, 4]. A number of *in vitro* studies have shown that ATP13A2 deficits can cause deficiencies in lysosomal, autophagic and mitochondrial functions, which are known characteristics of PD [2, 5–7]. *In vitro* studies have found that elevated ATP13A2 expression suppresses α-synuclein toxicity in multiple models, including rat midbrain primary dopamine neurons [2], implicating it as a potential target for PD therapeutics. Supporting this therapeutic possibility was the finding that surviving nigral dopamine neurons in patients with sporadic PD express *ATP13A2* mRNA at 5 to 10-fold higher levels than controls [1], although ATP13A2 protein levels show a more modest increase in these neurons [4]. In contrast, a separate study found ATP13A2 protein levels to be reduced in nigral dopamine neurons relative to controls [8] with a redistribution of the protein into α-synuclein-positive Lewy body inclusions [5, 8]. The divergent results from these human brain tissue studies may have resulted from examination of Lewy body disease cases with the coexisting age-related Azheimer-type pathologies of extracellular β-amyloid-positive plaques and/or tau-positive neurofibrillary tangles [9], as one cohort studied included both PD and DLB cases [4].

In this study, we sought to assess if ATP13A2 levels in Lewy body disease are modified by Alzheimer-type β-amyloid deposition by evaluating cases of pure PD that lack β-amyloid-positive plaques and pure dementia with Lewy bodies (DLB) and also β-amyloid-positive plaques. Such cases were examined for changes in and correlations between ATP13A2, α-synuclein and β-amyloid protein levels in cortical regions with and without Lewy bodies using Western blotting and ELISA. Changes in ATP13A2 cellular localization were also assessed using immunohistochemistry. To assess the earliest changes associated with α-synuclein aggregation, we evaluated regions displaying α-synuclein that do not undergo major neuron loss in PD.

## Results

### Increased Aβ42 levels in DLB compared with PD cases

Despite shorter disease durations for cases with DLB compared with PD (Table 1), ELISA results from the parahippocampal cortex show a 1.9-fold increase in relative membrane-associated Aβ42 in pure DLB over PD levels (p = 0.05), with a positive correlation between β-amyloid 1–42 (Aβ42) and α-synuclein levels (R = 0.66, p = 0.05). Aβ42 levels in pure DLB were increased 2.4-fold from controls (p = 0.02), but were not significantly different in PD compared to controls (p > 0.64). Protein levels were not related to age (p > 0.1) or postmortem delay (p > 0.3) in any group.

**Table 1:** **Demographic details for each cohort**

	Control	PD	DLB	***p*** value
**Sex (M:F)**	6:6	7:4	7:4	0.68^+^
**Age (years)**	77 ± 10 (62–92)	78 ± 6 (71–88)	78 ± 6 (67–88)	0.94^∆^
**PMD (hours)**	21 ± 11 (6–36)	13 ± 7 (3–23)	24.5 ± 12 (4–39)	0.07^∆^
**Duration (years)**	-	19 ± 8 (8–36)	7 ± 4 (1–13)	<0.001
**Braak PD stage** ^**#**^	-	9 IV; 2 V/VI	0 IV; 11 V/VI	<0.001^+^
**NIA Regan AD** ^*^	0/12	0/11	1/11	0.37^+^
**CDR**	0.0 ± 0.1 (0–0.5)	0.1 ± 0.2 (0–0.5)	2.4 ± 0.8 (1–3)^#^	<0.001^+^
**Aβ42 (μg/ml)** ^**^**^	13 ±8 (7.4-24.9)	16.5 ± 10 (7.0-31.1)	31.4 ± 11(17.8-42.3) ^#^	0.046^∆^

^#^[1].

^*^[3] None of the 11 PD cases and only 1 of the 11 DLB cases reached diagnostic criteria for Alzheimer’s disease, with neuritic beta-amyloid plaques and tau-positive neurofibrillary tangles present in this single DLB case. All other DLB cases displayed diffuse beta-amyloid plaques.

^^^Data obtained from parahippocampal cortices.

^#^Different from other groups on posthoc protected t test.

^+^Chi-square test, ^∆^Analysis of variance (ANOVA).

Data are presented as mean ± standard deviation (range) for post-mortem delay (PMD), age at death (Age), disease duration (Duration), clinical dementia rating scale (CDR) and β-amyloid 1–42 protein levels (Aβ42).

### Increases in α-synuclein and Aβ42 correlated with decreases in ATP13A2 levels

**Figure 1:**
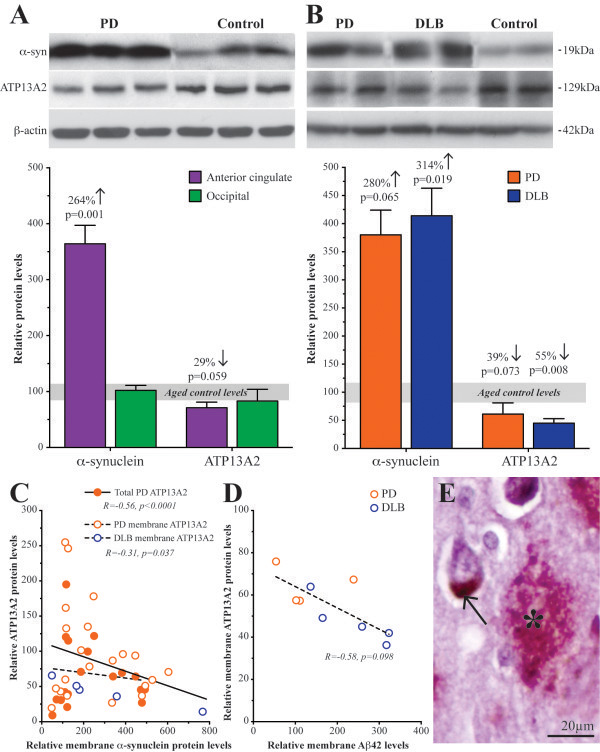
**Increases in α-synuclein and β-amyloid are correlated with decreases in ATP13A2 protein levels in Lewy body diseases. A** Representative Western immunoblots of α-synuclein, ATP13A2 and β-actin in membrane-associated protein fractions from PD and control anterior cingulate cortex. Quantitative data shows membrane-associated α-synuclein was increased 264% and total ATP13A2 was reduced 29% in PD anterior cingulate cortex, but neither α-synuclein nor ATP13A2 levels were significantly changed in PD occipital cortex. Data are shown as a percentage of mean control levels. **B** Representative Western immunoblots of α-synuclein, ATP13A2 and β-actin in membrane-associated protein fractions from PD, DLB and control parahippocampal cortex . Quantitative data shows membrane-associated α-synuclein was increased by 280% in PD and 314% in DLB compared with controls, and membrane-associated ATP13A2 was reduced from control levels by 39% in PD and 55% in DLB parahippocampal cortex. These protein changes were not significantly different between the PD and DLB cases in this region. Data are shown as a percentage of mean control levels. **C** Regression analysis revealed significant negative correlations between membrane-associated α-synuclein and both membrane-associated ATP13A2 and total ATP13A2 in anterior cingulate cortex in PD and DLB. Data are shown as a percentage of mean control levels. **D** Membrane-associated Aβ42 and ATP13A2 were significantly negatively correlated in PD and DLB parahippocamal cortex. Data are shown as a percentage of mean control levels. **E** Diffuse β-amyloid-positive plaques (asterisk) were present in DLB but not PD anterior cingulate cortex and showed no colocalization or physical association with ATP13A2-positive neurons (arrow).

We have previously shown that the most substantial change in α-synuclein is a shift from the soluble to the SDS-soluble membrane-associated fraction over the course of PD [10]. As expected, the levels of membrane-associated α-synuclein were significantly increased in the anterior cingulate but not occipital cortices compared with controls (264 ± 33% increase from control levels, p = 0.001; Figure 1A). In contrast, there was a reduction in the total level of ATP13A2 protein (soluble, membrane-associated and insoluble fractions) in PD anterior cingulate cortex compared with controls (29 ± 10% reduction from control levels, p = 0.059; Figure 1A). Similar changes were observed in the parahippocampal cortex, with increased levels of membrane-associated α-synuclein (280-314 ± 44-49% increase from control levels, p = 0.018) and reduced levels of membrane-associated ATP13A2 (39-55 ± 8-10% reduction from control levels, p = 0.009) in PD and DLB cases compared with controls (Figure 1B). These changes were not significantly different between the PD and DLB cases in this region (p > 0.71). Across all three regions examined (anterior cingulate, parahippocampal and occipital cortices), there was a significant positive correlation between membrane-associated ATP13A2 and total ATP13A2 levels (R = 0.67, p < 0.0001; Additional file 1: Figure S1A), while significant negative correlations were observed between membrane-associated α-synuclein and both membrane-associated (R = −0.31, p = 0.04) and total (R = −0.56, p < 0.0001) ATP13A2 levels in the PD, DLB and control cases (Figure 1C). Assessment of the anterior cingulate cortex alone strengthened the negative correlation between the level of ATP13A2 protein and increased membrane-associated α-synuclein protein levels (R = −0.77, p < 0.0001; Additional file 1: Figure S1B). There was also a negative correlation between Aβ42 and ATP13A2 levels in the PD and DLB cases in the parahippocampal cortex (R = −0.58, p = 0.098; Figure 1D). The more typical low levels of Aβ42 in the PD cases resulted in there being no correlation between ATP13A2 and α-synuclein levels in these samples (p = 0.28). ATP13A2 protein levels were not related to age (p > 0.49) or postmortem delay (p > 0.09) in any group.

### α-Synuclein and ATP13A2 both demonstrated increased insolubility and Lewy body staining

**Figure 2:**
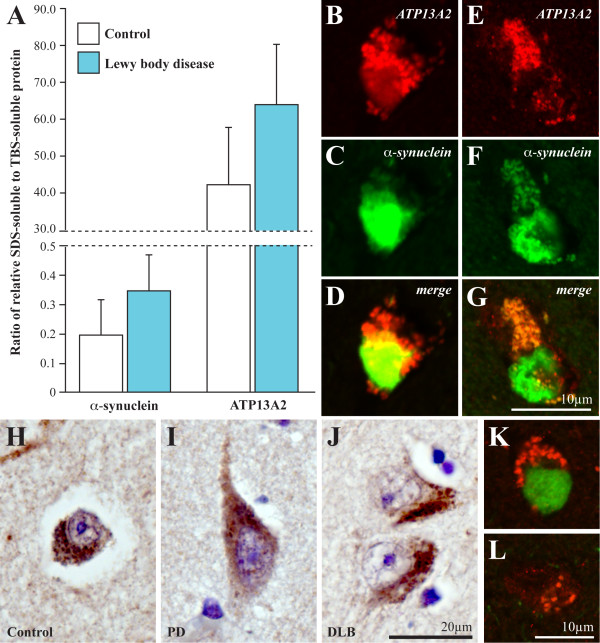
**A α-Synuclein and ATP13A2 both demonstrated increased insolubility in PD anterior cingulate cortex, with increased ratios of SDS-soluble to TBS-soluble protein. B-G** ATP13A2 (red; **B**, **E**) and α-synuclein (green; **C**, **F**) immunostaining in PD anterior cingulate cortex shows partial colocalization (yellow) in Lewy bodies in some (**D**) but not all (**G**) cortical neurons. **H-J** ATP13A2 immunolabeling of diffuse cytoplasmic puncta in anterior cingulate cortex was not different between neurons from control cases (**H**) and neurons without Lewy bodies from either pure PD cases (**I**) or pure DLB cases (**J**). **K-L** An increase in the number of ATP13A2 immunolabeled (red) cytoplasmic puncta was observed in neurons containing Lewy bodies (α-synuclein, green; **K**) compared to neurons without Lewy bodies (**L**) in anterior cingulate cortex of PD and DLB cases.

As α-synuclein is known to become increasingly insoluble during the formation of pathological Lewy bodies [10], assessment of the ratio of the more insoluble SDS compared with the more soluble TBS protein fractions was performed, with a ratio >1 indicating more insolubility and a ratio <1 indicating more soluble protein. As expected, in controls α-synuclein had an average ratio of <1 (0.196), confirming the normal predominance of soluble α-synuclein [10], while ATP13A2 had an average ratio of >1 (42.2), reflecting the multiple membrane spanning domains within the protein [10]. We identified increased insolubility in both α-synuclein (average ratio of 0.350) and ATP13A2 (average ratio of 64.2) in the anterior cingulate cortex of cases with Lewy bodies (Figure 2A), potentially reflecting the incorporation of ATP13A2 into insoluble Lewy bodies. Double-labeling immunoflourescence was then used to determine whether ATP13A2 protein co-localized with α-synuclein in Lewy bodies, with partial colocalization observed in some, but not all, cortical neurons (Figure 2B-G).

### Comparison of ATP13A2 cellular localization and protein expression in PD compared with DLB

Comparison of immunoperoxidase localization of ATP13A2 in anterior cingulate cortex between PD and DLB cases and controls was performed, as a recent study suggested increased ATP13A2 protein expression in a Lewy body disease cohort that included both PD and DLB cases [4]. Peroxidase immunolabeling confirmed the pattern of diffuse punctate staining within the cytoplasm of cortical neurons identified using immunoflourescence (Figure 2). The intensity of cytoplasmic immunostaining was variable within each case, but there were no overt differences between control neurons (Figure 2H) and non-Lewy body containing neurons in either PD or DLB (Figure 2I and J). However, an increase in the number of ATP13A2-immunolabeled cytoplasmic puncta was observed in cortical neurons containing Lewy bodies (Figure 2K) compared to those without (Figure 2L). Double-immunolabeling of ATP13A2 with β-amyloid in all cases confirmed that diffuse β-amyloid plaques were present in the DLB but not the PD or control cases, but revealed no colocalization or even any physical association between ATP13A2-positive neurons and β-amyloid-immunopositive plaques (Figure 1E).

## Discussion

In this study, we have identified that in cases with Lewy bodies there is a relationship between increasing α-synuclein levels and insolubility with an overall decrease in ATP13A2 levels but with the remaining ATP13A2 becoming more insoluble. The increase in ATP13A2 insolubility was associated with colocalization with α-synuclein in some Lewy bodies, as previously described [4, 5, 8]. We found no overt differences in ATP13A2 protein levels or cellular localization between pure PD and pure DLB cases. Our data is consistent with similar Western blot data from the substantia nigra of PD cases [5, 8], as well as with quantification of immunofluorescence staining showing decreased cytoplasmic ATP13A2 levels in substantia nigra neurons without pathology in cases with PD [5, 8]. The ten-fold increase in ATP13A2 mRNA expression previously demonstrated in isolated pigmented dopaminergic substantia nigra neurons from patients with PD [1] suggests that there may be either impaired translation or enhanced degradation of ATP13A2 protein. Enhanced protein degradation may be consistent with the partial rather than total overlap of ATP13A2 with the α-synuclein aggregations we observed in the cases with Lewy bodies. While it has previously been hypothesized that decreased ATP13A2 in the cytosol may be due to the protein becoming trapped in Lewy bodies, this does not seem likely considering the widespread nature of the decrease in ATP13A2 levels against the more limited number of neurons with Lewy bodies (see also [5, 8]). In the present study we observed more cytoplasmic ATP13A2 immunoreactive puncta in cortical neurons containing Lewy bodies than in neurons without Lewy bodies (see also [4]), suggesting that this change is more related to a change in the solubility of remaining protein. Overall our data suggest that in neurons with increasing α-synuclein insolubility, there is a corresponding loss of more soluble, and presumably functional, ATP13A2 protein levels, with an increase in visible aggregates containing both ATP13A2.

Several studies have shown that ATP13A2 deficiencies affect cellular functions known to be impaired in PD, notably lysosomal and mitochondrial mechanisms [2, 5–7]. *In vitro* studies have demonstrated that knockdown of ATP13A2 impairs lysosomal acidification, reducing lysosomal substrate degradation and clearance of autophagosomes [5], with impaired lysosomal degradation capacity resulting in α-synuclein accumulation and toxicity [11]. Increased mitochondrial mass and reactive oxygen species production, as well as decreased autophagic flux, has also been observed following ATP13A2 knockdown [6]. These lysosomal and mitochondrial deficits can be rescued by restoration of ATP13A2 levels, which supports earlier studies demonstrating that ATP13A2 can suppress α-synuclein toxicity in yeast models [2]. Our data is consistent with these *in vitro* studies, as excess abnormal α-synuclein in PD and DLB is associated with reduced ATP13A2. It remains unclear, however, whether accumulated α-synuclein causes reductions in ATP13A2 protein levels or if a decrease in functional ATP13A2 protein levels renders neurons vulnerable to α-synuclein toxicity in Lewy body diseases.

A recent study identified increased ATP13A2 immunoreactivity and protein levels in DLB frontal cortex [4]. However, closer examination of this study suggests that the DLB cases examined also had concomitant Alzheimer’s disease pathology. This may suggest that the increased ATP13A2 observed may be associated with the prevalent tau pathology observed in Alzheimer’s disease, as we found no evidence for an association with β-amyloid deposition. In support of this hypothesis, in the single DLB case with sufficient tau pathology to have coexisting Alzheimer’s disease we observed a similar increase in ATP13A2 immunostaining in cortical neurons, with occasional cortical neurons displaying cytoplasmic ATP13A2 aggregates, a finding associated with abnormal or reduced nuclear nucleic acid staining or neurofibrillary tangle-like structures (Additional file 1: Figure S2A). Double-labeling immunofluorescence in the same case confirmed colocalisation of ATP13A2 immunoreactivity in tau-immunopositive neurofibrillary tangles in some cortical neurons (Additional file 1: Figure S2B). This potential relationship between ATP13A2 and tau aggregation warrants further investigation to determine whether ATP13A2 may influence additional neurodegenerative processes. As ATP13A2 has a role in intracellular transport and vesicle trafficking [2, 12, 13], dysfunction of these processes in other neurodegenerative diseases may also impact on ATP13A2.

## Conclusions

We have identified that ATP13A2 protein levels decrease and become more insoluble in association with increased levels and insolubility of α-synuclein in cases with Lewy body diseases, and confirm that ATP13A2 is colocalized in some Lewy bodies. Evaluation of pure DLB cases with β-amyloid deposition revealed no ATP13A2 accumulation in association with these extracellular deposits. Because of the similarities in our observations between cases with pure PD and DLB, and because β-amyloid correlated with both α-synuclein and ATP13A2 protein levels (which correlated with each other), we suggest that ATP13A2 in pure Lewy body diseases is modulated mainly by the changes in α-synuclein rather than β-amyloid. In particular, the changes observed in ATP13A2 protein levels and localization in neurons containing α-synuclein pathology appear characteristic of Lewy body diseases. *In vitro* data show that ATP13A2 deficits lead to lysosomal, autophagic and mitochondrial dysfunction and α-synuclein accumulation, which are known features of Lewy body diseases, and that increasing ATP13A2 levels can alleviate α-synuclein toxicity [2, 12, 13]. Overall these data are supportive of the concept that increasing ATP13A2 levels may have potential therapeutic benefits in patients with Lewy body diseases (see [2]).

## Methods

### Brain tissue

Brain tissue from the anterior cingulate (Brodmann’s area (BA) 24/33) and/or parahippocampal (BA36/37/20) cortices (contain α-synuclein aggregates without substantial neuronal loss) and occipital association (BA18/19) cortex (does not accumulate α-synuclein and has no neuronal loss) of eleven autopsy-confirmed levodopa responsive, non-demented PD cases (nine Braak stage IV and two stages V/VI [14], no β-amyloid-positive plaques), eleven cases with pure DLB (all Braak stages V/VI, all with diffuse β-amyloid-positive plaques, one with coexisting Alzheimer’s disease pathology including neuritic β-amyloid-positive plaques and tau-positive neurofibrillary tangles [15]) and twelve age- and post-mortem delay-matched controls without neurological or neuropathological disease was obtained from the Sydney Brain Bank and New South Wales Tissue Resource Centre, who have institutional ethics approval to collect brain tissue for research purposes as part of the Australian Brain Bank Network. Dementia status was determined in longitudinal 1–2 year assessments using the Clinical Dementia Rating scale [16]. The study was approved by the Human Research Ethics Committee of the University of New South Wales and the Scientific Review Committee of the New South Wales Brain Banks. Demographic details of all cases are given in Table 1.

### Protein extraction

250 mg of fresh-frozen brain tissue from each region was available for protein extraction from ten controls, 9 PD and 6 pure DLB cases. Tissue was homogenized in TBS homogenization buffer (50 mM Tris, 125 mM NaCl, pH 7.4, 5 mM EDTA, 0.02% sodium azide) containing protease inhibitors (Complete, EDTA-free; Roche), followed by sonication (2 × 10sec bursts) and centrifugation at 120,000 g for 2 hr at 4°C, with supernatant collected as the TBS-soluble fraction containing cytosolic proteins. The pellet was resuspended in SDS solubilization buffer (TBS homogenization buffer containing 2% SDS), sonicated (2 × 10 sec bursts) and centrifuged at 100,000 g for 30 min at 25°C, with supernatant collected as the SDS-soluble fraction containing membrane-associated proteins. The remaining pellet was solubilized in 1 ml of SDS-urea solubilization buffer (TBS homogenization buffer containing 8% SDS and 8 M urea) by sonication, and collected as the detergent-insoluble urea-soluble fraction containing resolubilized protein aggregates. Protein concentration of all fractions was measured using the BCA assay (Pierce BCA Protein Assay Kit, Thermo Scientific 23225), according to manufacturer instructions. Samples were stored at −80°C.

### Western immunoblotting

TBS-soluble (soluble), SDS-soluble (membrane-associated) and urea-soluble (insoluble) fractions were assessed by Western immunoblotting for levels of ATP13A2 and α-synuclein protein, as previously described [10]. Briefly, protein samples were heated with sample buffer (2% SDS, 20% glycerol, 2.5% bromophenol blue, 12.5 mM Tris-HCl, pH 6.8, 5% 2-mercaptoethanol) and separated by reducing SDS-PAGE. Membranes were blocked in 5% skim milk dissolved in 1× TBS-T (0.87% NaCl, 0.01 M Tris, pH 7.4, with 0.1% Tween20) and incubated overnight in primary antibodies prior to detection with enhanced chemiluminescence (Western Enhance HRP Chemiluminescence Detection Kit, Millipore, or Amersham ECL Plus Western Blot Detection System, GE Healthcare) using horseradish peroxidase-conjugated secondary antibodies. Primary antibodies were specific for ATP13A2 (Sigma A9732 at 1:1000 dilution) and α-synuclein (BD Transduction Laboratories 610787 at 1:4000 dilution), with 14-3-3 (Santa Cruz Biotechnology sc-629 at 1:5000 dilution) or β-actin (Abcam ab6276 at 1:80,000 dilution) used as protein loading controls. Immunoblotting experiments were performed in duplicate. Relative levels of each protein were analyzed using Image J software (U. S. National Institutes of Health, Bethesda, USA). Intensity of each protein band was quantified and expressed as arbitrary units standardized to β-actin or 14-3-3 protein levels and an internal control sample for comparison. Multivariate statistical analyses were performed to identify differences in relative protein levels between the groups and regions, and Pearson correlations were used to identify related variables.

### Enzyme-linked immunosorbent assay (ELISA)

Levels of membrane-associated β-amyloid 1–42 (Aβ42) were measured in SDS-soluble PD and pure DLB protein fractions using a human Aβ42 ELISA kit (Invitrogen, KHB3441), according to the manufacturer’s instructions.

### Immunohistochemistry and immunofluorescence

Routine immunoperoxidase labeling of ATP13A2 was performed in 5 μm formalin-fixed paraffin-embedded tissue sections from the anterior cingulate cortex of four PD, four pure DLB, one DLB with concomitant Alzheimer’s disease and two control cases. Briefly, tissue sections were pretreated with citrate buffer, incubated overnight in ATP13A2 primary antibody (Sigma A9732 at 1:600 dilution) prior to incubation in biotinylated anti-rabbit secondary antibody and avidin-biotin tertiary antibody complex. ATP13A2 protein was visualized using 3, 3'-diaminobenzidine and sections counterstained with cresyl violet to identify cellular Nissl substance. Specificity of the immunolabeling was confirmed using a negative control section with primary antibody omitted, and specificity of the ATP13A2 antibody in human tissue sections was confirmed using a blocking peptide (see Additional file 1). Slides were assessed using brightfield microscopy.

Double-labeling immunofluorescence was performed to assess the level of colocalization of ATP13A2 protein with α-synuclein or β-amyloid in the anterior cingulate cortex of the same PD and DLB cases. Briefly, tissue sections were subjected to antigen retrieval with 90% formic acid and citrate buffer. For colocalization with α-synuclein, overnight incubation in a mixture of ATP13A2 and α-synuclein (BD Transduction Labs 610787 at 1:200 dilution) primary antibodies was performed with the proteins visualized using Alexa fluor-conjugated secondary antibodies (Molecular Probes, Life Technologies). These slides were assessed using confocal microscopy. For colocalization with β-amyloid, immunoperoxidase labeling for ATP13A2 was performed as described in the paragraph above, followed by overnight incubation in β-amyloid primary antibody (Covance SIG-39320 at 1:10,000 dilution) prior to secondary and tertiary antibody incubation, and visualization with NovaRED peroxidase substrate (Vector SK-4800). These slides were assessed using brightfield microscopy.

## Electronic supplementary material

Additional file 1: Figure S1: Significant correlations between ATP13A2 levels in different tissue protein fractions (**A**) and with the levels of α-synuclein (**B**). Methods for double-labeling immunofluorescence for ATP13A2 and phosphorylated tau; **Figure S2.** Increased ATP13A2 immunostaining was observed in a DLB case fulfilling pathological criteria for concomitant Alzheimer’s disease. Methods for the characterization of the ATP13A2 antibody; **Figure S3.** Western blotting and immunohistochemistry showing antibody specificity; **Figure S4.** Examples of the Western blots of ATP13A2 and α-synuclein levels compared with β-actin as the protein loading control in different tissue fractions from different regions in the different cohorts assessed. (PDF 9 MB)

## Abbreviations

Aβ42: β-amyloid 1–42
ANOVA: Analysis of variance
BA: Brodmann’s area
CDR: Clinical dementia rating scale
DLB: Dementia with Lewy bodies
HEK: Human embryonic kidney
PD: Parkinson’s disease
PMD: Post-mortem delay.
