# Supplementary material for: ATP13A2 (PARK9) protein levels are reduced in brain tissue of cases with Lewy bodies
Source: Acta Neuropathol Commun. 2013 May 9;1(1):11. doi: 10.1186/2051-5960-1-11 (PMC4046687; doi:10.1186/2051-5960-1-11)

SUPPLEMENTARY MATERIAL

Supplementary Figure 1 – Significant correlations between ATP13A2 levels in different tissue protein fractions (**A**) and with the levels of α-synuclein (**B**).

A The relative levels of membrane-associated ATP13A2 and total ATP13A2 protein were significantly positively correlated in PD anterior cingulate and occipital cortices. Data are shown as a percentage of mean control levels.

B The relative levels of total ATP13A2 and membrane-associated α-synuclein were significantly negatively correlated in PD anterior cingulate cortex. Data are shown as a percentage of mean control levels.

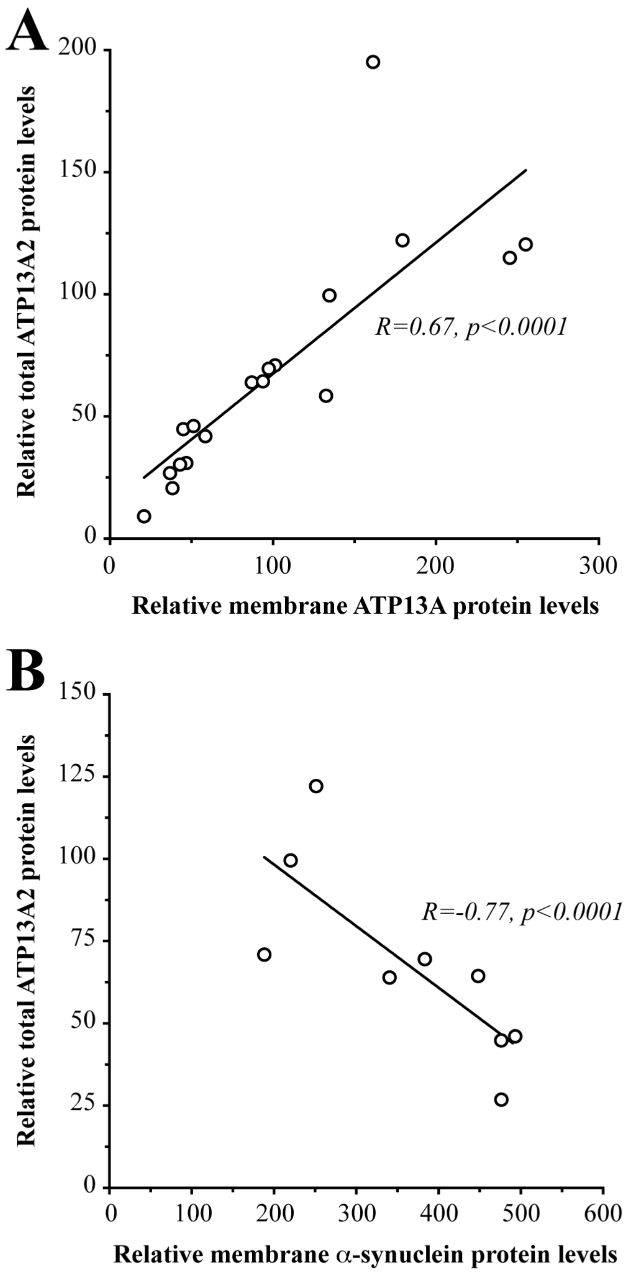

Double-labeling immunofluorescence for ATP13A2 and phosphorylated tau

Double-labeling immunofluorescence was performed, as described in Methods, to assess the level of colocalization of ATP13A2 and phosphorylated tau (Endogen MN-1020 at 1:10,000 dilution) in the anterior cingulate cortex of the single DLB case with sufficient tau pathology to have coexisting Alzheimer's disease. Some cortical neurons had tau-positive neurofibrillary tangles colocalizing ATP13A2 immunostaining (Supplementary Figure 2B).

Supplementary Figure 2 - Increased ATP13A2 immunostaining was observed in a DLB case fulfilling pathological criteria for concomitant Alzheimer's disease.

A Cytoplasmic ATP13A2-positive aggregates (closed arrows) associated with abnormal or reduced nuclear nucleic acid staining (asterisks) in some cortical neurons and with ATP13A2-positive neurofibrillary tangle-like aggregates (open arrow) in other cortical neurons.

B-D Some cytoplasmic ATP13A2-positive aggregates (red, **B**) were identified as tau-immunopositive neurofibrillary tangles (green, **C**) in cortical neurons. Merged image (yellow, **D**) shows colocalization.

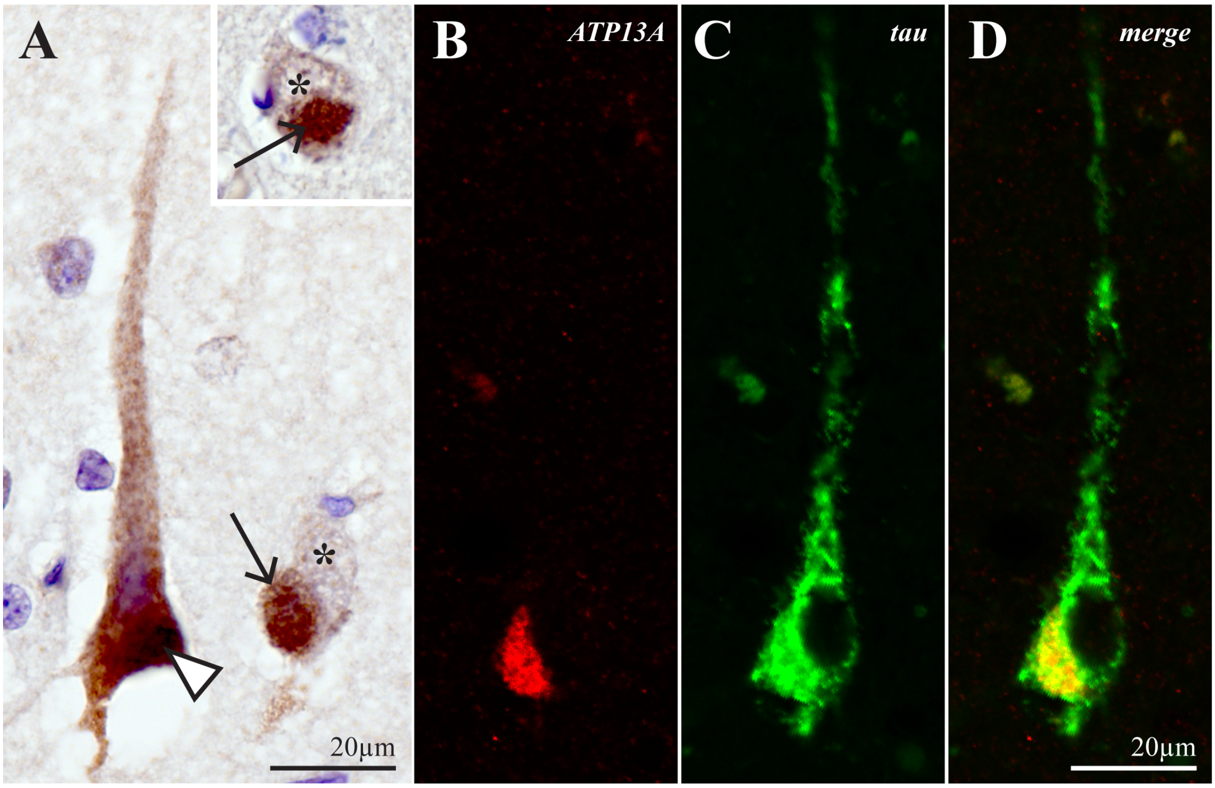

Characterization of the ATP13A2 antibody

The commercial ATP13A2 antibody (Sigma A9732) used in this study was characterized in human embryonic kidney (HEK293) cells over-expressing human ATP13A2. Western immunoblotting for ATP13A2 in protein extracted from non-transfected and transfected cells was performed using the commercial ATP13A2 antibody, as described. A single band recognizing full-length (129kDa) endogenous human ATP13A2 was observed in HEK cells (Supplementary Figure 3A, lane 1), with increased signal in cells over-expressing human ATP13A2 (Supplementary Figure 3A, lane 2). Incubating the ATP13A2 antibody with a specific human ATP13A2 C-terminal blocking peptide (United Biosystems) inhibited the ATP13A2 signal in a concentration-dependent manner, with excess peptide resulting in complete loss of ATP13A2 antibody signal (Supplementary Figure 3A, lane 3). Immunoperoxidase labeling of ATP13A2 in human brain tissue sections (Supplementary Figure 3B) was completely inhibited when the ATP13A2 antibody was incubated with an excess of blocking peptide (Supplementary Figure 3C), confirming the specificity of the ATP13A2 antibody in human brain tissue.

Supplementary Figure 3 – Western blotting and immunohistochemistry showing antibody specificity.

A Western immunoblotting of endogenous ATP13A2 (lane 1) and over-expressed human ATP13A2 (lane 2) in HEK293 cells identified full-length ATP13A2 signal, which was completely inhibited when an ATP13A2-specific blocking peptide was used (lane 3).

B, C Specificity of ATP13A2 immunostaining in human brain tissue sections (**B**) was confirmed using the same blocking peptide, which completely inhibited the APT13A2 signal (**C**).

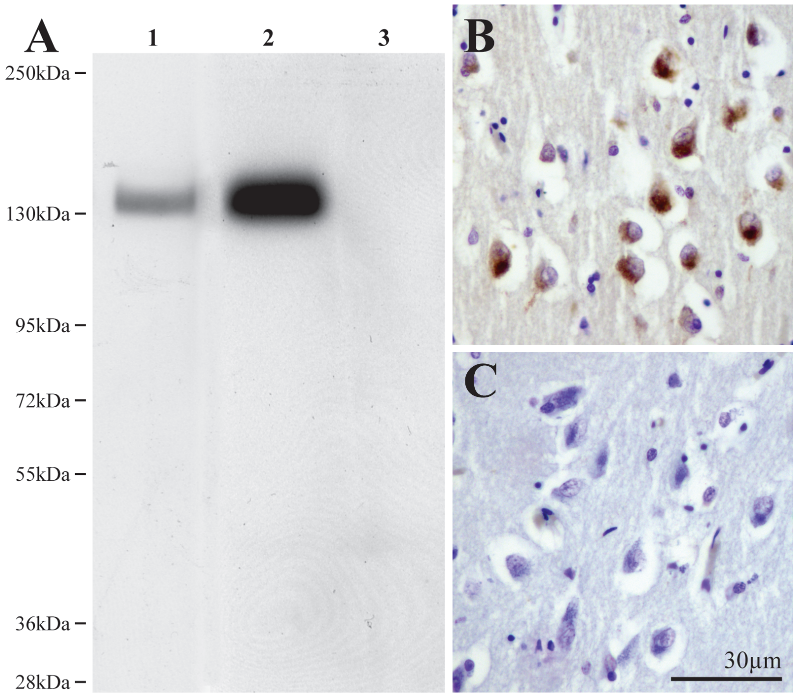

Supplementary Figure 4 – Examples of the Western blots of ATP13A2 and α-synuclein levels compared with β-actin as the protein loading control in different tissue fractions from different regions in the different cohorts assessed.

A Western blot images of PD (n=9 per region, marked with asterisks) and control (n=10 per region) cases assessed with ATP13A2 in anterior cingulate and occipital cortices. ATP13A2 was assessed in TBS-soluble, SDS-soluble and urea-soluble fractions, with β-actin or 14-3-3 used as a protein loading control.

B Western blot images of PD (n=9 per region, marked with asterisks) and control (n=10 per region) assessed with α-synuclein in anterior cingulate and occipital cortices. α-Synuclein was assessed in TBS-soluble, SDS-soluble and urea-soluble fractions, with β-actin used as a protein loading control.

C Western blot images of PD and DLB cases (n=9, marked with asterisks) and control cases (n=3) assessed with ATP13A2 and α-synuclein in the SDS fraction of the parahippocampal cortex, with β-actin used as a protein loading control.

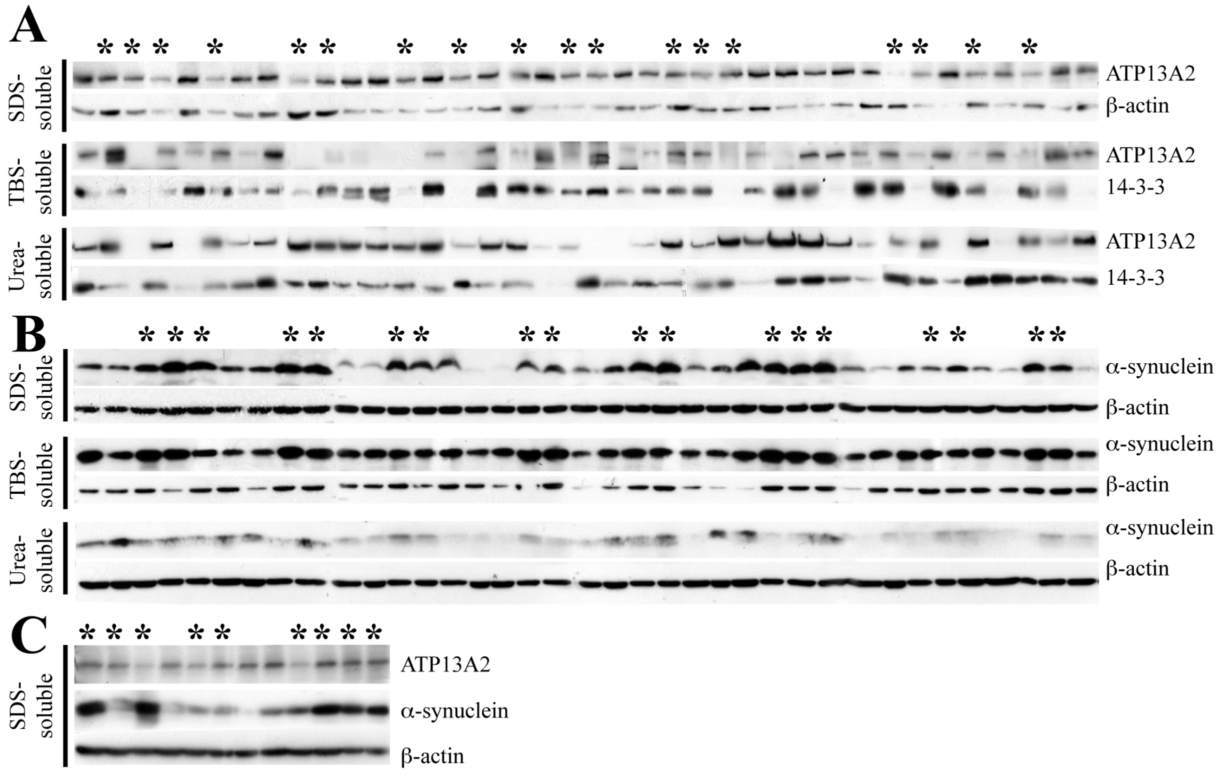

Supplement: Supplementary file 1 — Additional file 1: Figure S1: Significant correlations between ATP13A2 levels in different tissue protein fractions (A) and with the levels of α-synuclein (B). Methods for double-labeling immunofluorescence for ATP13A2 and phosphorylated tau; Figure S2. Increased ATP13A2 immunostaining was observed in a DLB case fulfilling pathological criteria for concomitant Alzheimer’s disease. Methods for the characterization of the ATP13A2 antibody; Figure S3. Western blotting and immunohistochemistry showing antibody specificity; Figure S4. Examples of the Western blots of ATP13A2 and α-synuclein levels compared with β-actin as the protein loading control in different tissue fractions from different regions in the different cohorts assessed. (PDF 9 MB) [file 40478_2013_6_MOESM1_ESM.pdf]
